# Exploring the association between metal(loid)s and human semen quality: a preliminary case study in a petrochemical complex

**DOI:** 10.1007/s11356-025-37173-x

**Published:** 2025-11-14

**Authors:** Elena Sánchez-Resino, Ana González-Ruiz, Jordi Sierra, Carlos Martínez-Pinto, María Fernández de la Puente, Nadine Alkhoury, María Ángeles Martínez, Nancy Babio, Albert Salas-Huetos, Jordi Salas-Salvadó, Rubén Gil-Solsona, Pablo Gago-Ferrero, José L. Domingo, Montse Marquès

**Affiliations:** 1 Laboratory of Toxicology and Environmental Health, Faculty of Medicine and Health Science, Universitat Rovira i Virgili, Reus, Spain; 2 Institut d’Investigació Sanitària Pere Virgili, Reus, Spain; 3 Facultat de Farmàcia i Ciències de L’Alimentació, Universitat de Barcelona, Barcelona, Spain; 4 Institute of Environmental Assessment and Water Research, IDAEA-CSIC, Barcelona, Spain; 5 Universitat Rovira i Virgili , Unitat de Nutrició Humana, Departament de Bioquímica i Biotecnologia, Alimentació, Nutrició, Desenvolupament i Salut Mental (ANUT-DSM), Reus, Spain; 6 CIBER de Fisiopatología de La Obesidad y Nutrición, Instituto de Salud Carlos III, Madrid, Spain; 7 Department of Pharmacology, Therapeutics and Toxicology, Faculty of Veterinary, Universitat Autònoma de Barcelona, Cerdanyola del Vallès, Spain; 8 Unitat de Medicina Preventiva i Bioestadística, Departament de Ciències Mèdiques Bàsiques, Alimentació, Nutrició, Desenvolupament i Salut Mental (ANUT-DSM), Universitat Rovira i Virgili, Reus, Spain

**Keywords:** Metals, Metal(loid)s, Trace elements, Male fertility, Semen quality, Petrochemical industry

## Abstract

Occupational exposure to metal(loid)s in petrochemical industries has raised concerns about possible effects on male reproductive health. This study examines the link between metal(loid) exposure and semen quality parameters among petrochemical workers (PWs) compared to a non-petrochemical control group, using the WHO 2021 reference values as a standard. A cross-sectional analysis was performed on 176 healthy individuals, consisting of 22 PWs and 154 controls. Blood levels of arsenic (As), chromium (Cr), lead (Pb), tin (Sn), and thallium (Tl) were analyzed, and semen quality parameters, such as sperm concentration, total sperm count, motility, morphology, vitality, and volume, were evaluated and considered the main outcomes of the study. Overall, concentrations and detection frequencies were relatively low, indicating a scenario of low-level exposure. Petrochemical workers showed lower blood concentrations of Cr, Sn, and Pb compared to controls. Detection frequencies were higher in the petrochemical group than in controls (*p* > 0.05). Semen quality analysis revealed that PW had non-significant (*p* > 0.05) higher total sperm count, normal morphology, and vitality and statistically significant (*p* < 0.05) higher sperm concentration than controls. On the contrary, total motility and volume were non-significantly lower in PWs than in controls. Correlation analysis confirmed significant associations among semen parameters and identified positive associations between Pb and total motility (*ρ* = 0.24, *p* < 0.01) and Cr and Sn (*ρ* = 0.38, *p* < 0.001). In turn, Pb showed a positive association with total motility (*ρ* = 0.24, *p* = 0.001), abstinence (*ρ* = 0.17, *p* < 0.05), sperm concentration (*ρ* = 0.16, *p* < 0.05), and sperm count (*ρ* = 0.17, *p* < 0.05). Additionally, linear and logistic regression models demonstrated a significant positive association between blood Pb and total motility (β 3.58%, CI 1.01–6.15; OR 0.73%, CI 0.13–1.59). The results of the present study highlight the complexity of metal(loid) exposures and suggest that lifestyle and other environmental factors may influence semen quality. Longitudinal studies are warranted to better understand these associations.

**Supplementary Information:**

The online version contains supplementary material available at 10.1007/s11356-025-37173-x.

## Introduction

Metals and metalloids (metal(loid)s) encompass a diverse group of chemicals found in both natural and anthropogenic forms. They are encountered through various sources, including conventional agricultural practices and industrial activities, particularly those involving the combustion of fossil fuels (Marquès et al. 2017). Some of these elements exhibit endocrine-disrupting properties, which have been linked to disruptions in hormone-dependent metabolic processes in various animal species (Copat et al. 2013; Sobolewski et al. 2018) and to increased risks of respiratory diseases, cancer, diabetes, and infertility in humans (Marquès et al. 2020). According to the WHO, infertility is a condition experienced by one in six people at some point in their lives. Despite extensive research, there is a notable scarcity of studies focused on male fertility (WHO 2023). Existing literature connects metal(loid) exposure with reproductive health concerns and the rising incidence of infertility.

Arsenic (As) is a ubiquitous toxic metalloid. Notably, As(III) is more toxic than As(V) and has been linked to testicular tissue damage and compromised semen quality (Anyanwu and Orisakwe 2020). The cytotoxic potential of As_2_O_3_ on Sertoli cells has been associated with reduced cell viability and increased cell death in cultures (Zheng et al. 2024). However, its precise role in the male reproductive system remains only partially understood (Bhardwaj et al. 2021).

Chromium (Cr) is a trace element essential for normal glucose and lipid metabolism and is naturally present in the human body. However, Cr is widely released into the environment due to industrial activities, particularly through its use in metal plating and leather tanning and as a component in various alloys (ATSDR (Agency for Toxic Substances and Disease Registry) 2020; Vinceti et al. 2018). While necessary in trace amounts, elevated concentrations of Cr—especially in its hexavalent form, Cr(VI)—can be toxic and pose significant health risks. High levels of Cr have been linked to various reproductive health issues, including decreased sperm quality and abnormalities in sperm morphology, such as teratozoospermia and asthenozoospermia (Hossini et al. 2022; David et al. 2022).

Beyond its well-known neurotoxicity, lead (Pb) has also been associated with fertility issues (Kumar et al. 2020). Some studies have found a significant correlation between Pb concentration in seminal plasma and lower sperm counts (Wijesekara et al. 2020). Pb may inhibit spermatogenesis by causing structural changes in the intracellular junctions of the seminiferous tubule and by altering germ cells and Sertoli cells (El Shafai et al. 2011). It can also modify the patterns of expression of PGC-1alfa, which is essential for proper mitochondrial function (Ramos-Treviño et al. 2018). However, the findings of Tuncay et al. (2021) do not support this association, as Pb concentration in seminal plasma was not found to be associated with seminal quality.

Tin (Sn) is a metal commonly used in the production of various consumer goods, including coatings, plastics, and food packaging materials. While low levels of Sn are generally considered harmless, high exposure—particularly to organotin compounds—has been linked to adverse health effects, including reproductive toxicity (Ghaffari and Motlagh 2011). Studies have shown that organotin compounds can disrupt endocrine function, leading to alterations in hormone levels and negatively impacting sperm quality (Graceli et al. 2013). Additionally, Sn exposure has been associated with oxidative stress and inflammatory responses in the male reproductive system, which may contribute to impaired spermatogenesis and reduced semen quality (Omu 2013). Despite its widespread use, research on the specific impacts of Sn on male fertility remains limited, highlighting the need for further studies on its potential reproductive health risks.

Thallium (Tl) is a highly toxic metal with no known biological role, commonly encountered as a byproduct of industrial processes such as coal combustion, cement production, and metal smelting (Fisher and Gupta 2024). Animal studies have reported that Tl exposure can lead to reduced sperm motility, abnormal sperm morphology, and disruptions in testosterone levels, oxidative stress, and mitochondrial function (Formigli et al. 1986; Hanzel and Verstraeten 2006; Anaya-Ramos et al. 2021), which may contribute to impaired spermatogenesis and compromised semen quality. Despite its toxicity, research on Tl’s specific effects on male fertility remains sparse, underscoring the importance of further investigation into its reproductive health implications.

Living near petrochemical industries or working within these complexes can lead to exposing individuals to higher levels of metal(loid)s than the general population (Jia et al. 2022; Calogero et al. 2021; Shi et al. 2021; Branch et al. 2021). Numerous studies have assessed metal(loid) levels in areas surrounding petrochemical complexes, identifying them as hot spots for metals like Cr, Pb, V, As, and Ni, and have further evaluated their potential health impacts on local populations. Notably, one of the largest petrochemical complexes in southern Europe is in the province of Tarragona (Catalonia, Spain). Previous studies have reported metal concentrations in the surrounding areas below the safety threshold, with health risk assessments suggesting low health risks for the population (Kou et al. 2023; Nadal et al. 2011). However, research on the specific health impacts of metal(loids) on the local population remains limited (Marquès et al. 2020; Rovira et al. 2014; Vallecillos et al. 2024), with a notable lack of studies focused on their effects on male fertility.

The present study is aimed at evaluating whether working in the petrochemical complex of Tarragona (Catalonia, Spain) is associated with elevated exposure to metal(loid)s, potentially leading to adverse effects on semen quality. The study leverages blood and semen samples from adult men (18–40 years old) within the lifestyle and environmental determinants of the seminogram and other male fertility–related parameter (Led-Fertyl) cohort. Arsenic, Cr, Pb, Sn, and Tl were included in this investigation based on their reported occurrence near petrochemical industries in the area, as well as their potential reproductive toxicity (Calogero et al. 2021; Goutam Mukherjee et al. 2022). Blood was chosen as the most suitable biological matrix to assess the chronic exposure to these elements because of their accumulation in erythrocytes (Gil and Hernández 2015; Martínez-Morata et al. 2023). Furthermore, we extended our analysis to assess the relationship of these metal(loid) concentrations on semen quality parameters, including semen volume, sperm concentration, total sperm, total motility, live sperm, and normal-shaped sperm.

## Materials and methods

### Population and data collection

**Fig. 1:**
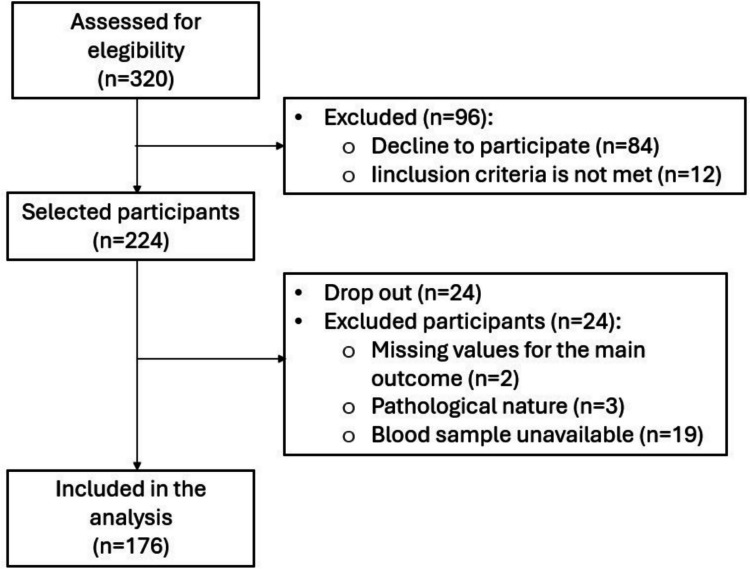
Flow chart

The recruitment of Led-Fertyl participants took place at the University Hospital Sant Joan de Reus (Reus, Catalonia, Spain). Up to 320 healthy men aged 18–40 were invited to participate. A total of 96 participants were excluded because they declined to participate (*n* = 84) or did not meet the inclusion criteria (*n* = 12). Hence, 224 individuals were selected as participants. Subsequently, information on sociodemographic and lifestyle was collected through in-person and online questionnaires, as previously described (Valle-Hita et al. 2024). These data were anonymously integrated into the Led-Fertyl database following data protection law. Twenty-four out of the 224 subjects dropped out of the study. Of the 200 individuals for the present analysis, 24 were discarded because there were missing values for the main outcome (*n* = 2), because of the undiagnosed pathological nature of the semen quality parameters (*n* = 3), or because blood samples had not been collected yet (*n* = 19), leading to a final cohort of 176 participants (Fig. 1). This study was performed in line with the principles of the Declaration of Helsinki. Both online and written informed consent were signed and provided by all participants. Approval was granted by the Ethics Committee of the Pere Virgili Health Research Institute (2019/No.: 181/2019).

The participants (*n* = 176) were divided into two groups: petrochemical workers (PWs) (*n* = 22) and non-petrochemical workers (control) (*n* = 154) to evaluate whether employment in the petrochemical industry increases metal(loid) concentrations in blood, potentially impacting semen quality.

Each participant provided a semen sample in a 100-mL sterile polypropylene pot after 3–7 days of complete sexual abstinence, as already detailed elsewhere (Martínez et al. 2024). Semen analysis was performed using the computer-assisted sperm analysis (CASA) SCA® system version 6.5.0.67 (Microptic) and an Olympus CX43 phase contrast microscope (EVIDENT Corporation) and evaluated according to the WHO’s guidelines to assess the following parameters: semen volume, pH, vitality, motility, morphology, count, and concentration (WHO 2021).

### Metal(loid) determination

Venous blood samples were collected after a minimum fasting period of 8 h using 10 mL Vacutainer tubes containing heparin/lithium anticoagulant. A 2 mL aliquot of blood was then separated and preserved in a cryovial at − 20 °C. Sample digestions were conducted using a microwave digestion system. Briefly, 0.5 mL of H_2_O_2_ (30% in Milli-Q water), 0.5 mL of HNO3 (65% in Milli-Q water; Suprapur E. Merck, Darmstadt, Germany), and 0.2 mL of the blood sample were mixed in a microinsert. The microwave temperature was set at 200 °C and held for 20 min, and then, the samples were cooled to room temperature. The resulting solution was diluted to 25 mL with Milli-Q water and stored at − 20 °C until analysis by inductively coupled plasma-mass spectrometry (ICP-MS) (Perkin-Elmer, NexIon 350D).

### Quality control

To minimize the contamination risk, all materials used for blood sample preservation and digestion were soaked overnight in HNO_3_ (10% in Milli-Q water) and then rinsed with deionized water before use. Samples were analyzed in duplicate, with a procedural blank and a standard (reference Seronorm Trace Elements Whole Blood L-2 (SERO AS)) included in each batch of ten samples. Recoveries ranged between 80 and 140%, with LODs set at 6.25 ng/mL for Sn and 12.5 ng/mL for As, Cr, Pb, and Tl.

### Statistical analyses

The Led-Fertyl database (May 2023) was used for statistical analyses. Led-Fertyl demographic, anthropometric, and lifestyle characteristics are reported as mean (standard deviation) and median (minimum, maximum). Firstly, the Kolmogorov–Smirnov test was applied to determine whether our data follow a normal distribution. Since the data did not meet the assumption of normality, the Mann–Whitney *U* test was used to compare occupational groups (petrochemical workers vs. controls). Fisher’s exact test was employed to evaluate differences in detection frequencies of metal(loid)s and sperm quality parameters exceeding WHO reference limits according to the occupational groups. Additionally, Spearman’s correlation coefficients were calculated to assess the strength and direction of the monotonic relationships between demographic factors, anthropometric and lifestyle factors, metal(loid) concentrations, and semen quality parameters.

Linear regression models were applied to estimate the association between Pb (the metal showing the highest detection frequency) and semen quality parameters (concentration, normal form, sperm count, total motility, vitality, volume). Because of the non-normal distribution of the data, Pb levels were log-transformed and analyzed as a continuous variable. Results were reported as β-coefficients and their 95% confidence intervals (CIs). Additionally, logistic regression models were fitted to assess the association between Pb log-transformed concentrations and the likelihood of having the semen quality parameters below the WHO lower limit (WHO 2021). Results are reported as odds ratios (ORs) and their 95% confidence intervals (CIs). Both linear and logistic regression models were adjusted by the following confounders: age (years), body mass index (kg/m^2^), physical activity (MET min/week), and abstinence period (number of days).

All statistical analyses were conducted using RStudio (RStudio 2023). LOD/2 was imputed to the non-detected observations. The level of statistical significance was established at 0.05 to assess the results.

## Results

### Descriptive analysis

Demographic, anthropometric, and lifestyle characteristics, blood metal(loid) levels, and semen quality parameters for both PW and control groups are summarized in Table 1. Detailed results per participant are provided in the Supporting Information (SI, Table S1). In brief, petrochemical workers were older (PWs = 32.1 ± 3.7 years vs. controls = 28.3 ± 5.6 years), had a slightly higher BMI (PWs = 25.1 ± 2.32 kg/m^2^ vs. controls = 24.4 ± 3.26 kg/m^2^), and reported lower levels of physical activity (PWs = 3500 ± 1660 METs/week vs. controls = 4090 ± 3145 METs/week) compared to participants with different employment.

**Table 1:** Demographic and lifestyle characteristics, metal(loid)s, and semen quality of Led-Fertyl participants

	Overall (*n* = 176)	Petrochemical workers (*n* = 22)	Control (*n* = 154)	*p*-value
Age (years)				**0.001**
Mean (SD)	28.8 (5.5)	32.1 (3.7)	28.3 (5.6)	
Median [min, max]	28.0 [18.0, 40.0]	32.0 [24.0, 37.0]	28.0 [18.0, 40.0]	
BMI (kg/m^2^)				0.214
Mean (SD)	24.5 (3.2)	25.1 (2.32)	24.4 (3.26)	
Median [min, max]	24.4 [17.4, 36.5]	24.7 [21.8, 30.5]	24.3 [17.4, 36.5]	
Physical activity (METs/week)				0.794
Mean (SD)	4016 (3003)	3500 (1660)	4090 (3145)	
Median [min, max]	3584 [280, 23357]	3400 [420, 6730]	3627 [280, 23400]	
Abstinence (days)				0.285
Mean (SD)	2 (0.84)	2 (0.79)	2 (0.85)	
Median [min, max]	1 [1, 5]	2 [1, 4]	2[1, 5]	
Blood metal(loid)s (ng/mL)				
As				0.598
Mean (SD)	6.83 (2.63)	–	6.83 (2.81)	
DF	7 (4.0%)	0 (0%)	7 (4.5%)	
Cr				0.118
Mean (SD)	7.29 (8.36)	4.99 (6.36)	7.23 (8.72)	
DF	5 (2.8%)	2 (9.1%)	3 (1.9%)	
Pb				1
Mean (SD)	16.2 (23.6)	9.87 (10.9)	16.2 (24.9)	
DF	54 (30.7%)	7 (31.8%)	47 (30.5%)	
Sn				0.648
Mean (SD)	4.03 (3.85)	2.85 (4.36)	3.94 (3.85)	
DF	12 (6.8%)	2 (9.1%)	10 (6.5%)	
Tl				–
Mean (SD)	–	–	–	
DF	0 (0%)	0 (0%)	0 (0%)	
Semen quality parameters				
Sperm concentration (× 10^6^)				**0.032**
Mean (SD)	64.7 (53.6)	91.9 (66.4)	60.7 (50.3)	
Median [min, max]	49.3 [0, 288]	72.9 [10.1, 220]	48.2 [0, 288]	
Normal form (%)				0.549
Mean (SD)	10.8 (7.51)	12.2 (8.86)	10.6 (7.33)	
Median [min, max]	9.5 [0, 39.5]	10.8 [1.00, 30.5]	9.5 [0, 39.5]	
Sperm count (× 10^6^)				0.091
Mean (SD)	221 (186)	305 (243)	209 (173)	
Median [min, max]	169 [0, 1023]	251 [35.1, 826]	159 [0, 1023]	
Total motility (%)				0.428
Mean (SD)	60.2 (16.8)	59.1 (11.6)	60.3 (17.3)	
Median [min, max]	61.7 [0, 91.9]	57.9 [39.0, 77.2]	62.0 [0, 91.9]	
Vitality (%)				0.928
Mean (SD)	78.5 (12.5)	79.2 (10.0)	78.5 (12.5)	
Median [min, max]	80.5 [0, 96]	80.5 [43.0, 92.0]	80.5 [0, 96]	
Volume (mL)				0.8
Mean (SD)	3.63 (1.41)	3.58 (1.50)	3.65 (1.41)	
Median [min, max]	3.50 [0.60, 9.40]	3.50 [1.10, 6.70]	3.50 [0.60, 9.40]	

*SD* standard deviation, *Min* minimum, *Max* maximum, *BMI* body mass Index, *DF* detection frequency

Values in bold indicate statistical significance (*p*-value < 0.05)

Arsenic, Cr, Pb, and Sn were detected in at least five out of the 177 individuals, while Tl was not detected in any participant. Lead was the most frequently detected metal, found in 31.8% of PWs and 30.5% of controls. Chromium and Tl were identified in 9.1% of PWs and in 9.1% and 6.5% of controls, respectively. Arsenic was not detected in any individual from PWs but was present in 4.5% of the controls. Despite relatively low detection frequencies overall, indicating a scenario of low-level exposure in both groups, the occurrence of certain elements is noteworthy. Interestingly, the mean concentrations of Cr (4.99 ± 6.36 ng/mL in PWs vs. 7.23 ± 8.72 ng/mL in controls), Pb (9.87 ± 10.9 ng/mL in PWs vs. 14.1 ± 25.8 ng/mL in controls), and Sn (2.85 ± 4.36 ng/mL in PWs and 3.94 ± 3.85 ng/mL in controls) were slightly lower among petrochemical workers than in controls. Although the detection frequencies of Cr, Pb, and Sn were marginally higher in the petrochemical group, no statistically significant differences were observed between occupational groups (*p* > 0.05). Due to these low detection rates, no comparative statistical tests on concentrations were performed.

Total motility and volume were slightly lower among petrochemical workers (59.1 ± 11.6% and 3.58 ± 1.50 mL) compared to the control group (60.3 ± 17.3% and 3.65 ± 1.41 mL), though these differences were not statistically significant (*p* > 0.05). In contrast, petrochemical workers had higher values for sperm concentration (91.9 ± 66.6 million/mL) compared to men in other occupations (60.7 ± 50.3 million/mL), normal morphology (12.2 ± 8.86% vs. 10.6 ± 7.33%), total sperm count (305 ± 243 million/ejaculate vs. 209 ± 173 million/ejaculate), and vitality (79.2 ± 10% vs. 78.5 ± 12.5%), respectively, with sperm concentration being the only parameter to reach statistical significance (*p* < 0.05).

Table 2 presents key semen quality parameters according to the WHO 2021 guidelines (WHO 2021), alongside the number of participants below the reference values in the different exposure groups (PWs and controls). Overall, the number of participants below the lower reference limits was as follows: 16 (9.1%) for sperm concentration, 27 (15.3%) for normal morphology, 13 (7.4%) for total sperm count, 26 (14.8%) for total motility, 9 (5.1%) for vitality, and 4 (2.3%) for volume. Although the absolute number of individuals below the WHO reference values varies between petrochemical workers and controls due to the differences in the sample size, the prevalence remains similar. An exception is total motility, which was more frequently below the reference value in controls (16.1%) compared to petrochemical workers (9%). No statistically significant differences were reported when comparing the detection frequencies below the WHO reference limits between occupational groups (*p* > 0.05).

**Table 2:** Semen quality parameters according to the WHO 2021 guidelines (WHO 2021) along with the absolute (n) and relative (%) number of participants below the reference value

Parameter	Reference value (lower limit)	Overall n(%) < the reference value (*n*=176)	PW n(%) < the reference value (*n*=22)	Control n(%) < the reference value (*n*=154)	*p*-value
Sperm concentration	≥ 15 million/mL	16 (9.1%)	2 (9.1%)	14 (9.1%)	1
Normal sperm morphology	≥ 4%	27 (15.3%)	4 (18.2%)	23 (14.9%)	0.752
Total sperm count	≥ 39 million/ejaculate	13 (7.4%)	1 (4.5%)	12 (7.8%)	1
Total motility	≥ 42%	26 (14.8%)	2 (9.0%)	24 (15.6%)	0.538
Vitality	≥ 54%	9 (5.1%)	1 (4.5%)	8 (5.2%)	1
Volume	≥ 1.4 mL	4 (2.3%)	1 (4.5%)	3 (1.9%)	0.417

Among the individual semen quality parameters (Supporting Information, Table S1), 61 participants (34.7%) had at least one parameter below the respective reference value. Of these, 5 (22.7%) were petrochemical workers, and 56 (36.4%) were from the control group. Additionally, 4 participants (1 petrochemical worker (4.5%) and 3 controls (1.9%)) exhibited values below the reference limits for sperm concentration, normal form, sperm count, total motility, and vitality.

### Correlation analysis of sociodemographic and lifestyle factors, metal(loid) concentrations, and semen quality parameters

**Fig. 2:**
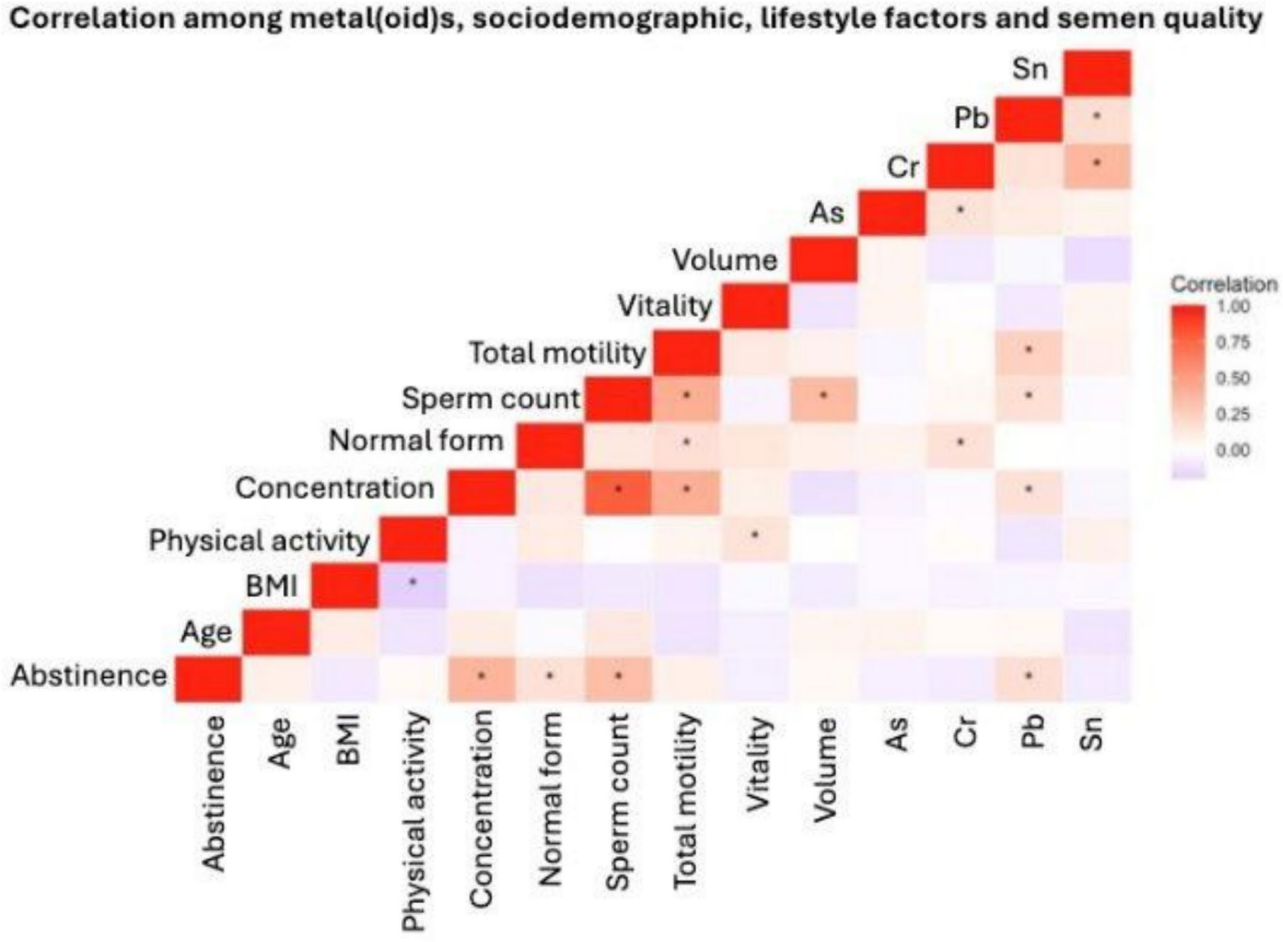
Heat map illustrating correlations among metal(loid) concentrations, sociodemographic and lifestyle factors, and semen quality parameters. The asterisk (*) represents that the correlation is statistically significant (*p* < 0.05)

A heat map was constructed to visualize the correlations between metal(loid) concentrations (As, Cr, Sn, Pb); sociodemographic, anthropometric, and lifestyle factors (age, BMI, physical activity, abstinence period); and semen quality parameters (sperm concentration, normal morphology, total sperm count, total motility, vitality, and semen volume) (Fig. 2). All correlation coefficients (*ρ*) and statistical significance (*p*-value) are included in the Supporting Information (Supporting Information, Table S2). Chromium and Sn were positively correlated with each other (*ρ* = 0.38, *p* < 0.001), while other elements, like Pb and Sn (*ρ* = 0.17, *p* < 0.05) and Cr and Sn (*ρ* = 0.15, *p* < 0.05), showed weak, but statistically significant, correlations. In turn, Pb showed a positive association with total motility (*ρ* = 0.24, *p* = 0.001), abstinence (*ρ* = 0.17, *p* < 0.05), sperm concentration (*ρ* = 0.16, *p* < 0.05), and sperm count (*ρ* = 0.17, *p* < 0.05). Additionally, Cr showed a positive correlation with sperm normal morphology (*ρ* = 0.16, *p* < 0.05). The rest of the correlations remained weak and without statistical significance (*p* > 0.05).

### Association between Pb and semen quality

Table 3 presents the multiple β-coefficients and odds ratios (ORs), along with the respective 95%CIs, for the associations between continuous blood log-transformed Pb concentrations and semen quality parameters. Log-transformed Pb concentration was negatively associated with semen volume (β − 0.02, CI − 0.24–0.19; OR − 0.35, CI: − 1.43–0.93). However, this association was not statistically significant (*p* > 0.05). Similarly, non-statistically significant (*p* > 0.05) positive associations were observed between log-transformed Pb concentrations and sperm concentration (β 1.84, CI − 5.43–9.11; OR 0.74, CI − 0.02–1.92), the percentage of sperm with normal morphology (β 0.09, CI − 1.07–1.26; OR 0.37, CI − 0.11–0.96), the total sperm count (β 22, CI − 3.17–48; OR 0.51, CI − 0.18–1.61), and the percentage of vitality (β 0.12, CI − 1.95–2.18; OR 0.23, CI − 0.45–1.14). Contrary to expectations, log-transformed Pb concentrations showed a positive and statistically significant (*p* < 0.05) association with the percentage of total motility (β 3.58, CI 1.01–6.15; OR 0.73%, CI 0.13–1.59).

**Table 3:** Multiple-adjusted β-coefficients and OR, including their 95%CI for seminogram parameters across blood log-transformed Pb concentrations

	β-coefficients (95%CI)	OR (95%CI)
Sperm concentration (× 10^6^)	1.84 (− 5.43, 9.11)	0.74 (− 0.02, 1.92)
Normal sperm morphology (%)	0.09 (− 1.07, 1.26)	0.37 (− 0.11, 0.96)
Total sperm count (× 10^6^)	22 (− 3.17, 48)	0.51 (− 0.18, 1.61)
Total motility (%)	**3.58 (1.01, 6.15)**	0.73 (0.13, 1.59)
Vitality (%)	0.12 (− 1.95, 2.18)	0.23 (− 0.45, 1.14)
Volume (mL)	− 0.02 (− 0.24, 0.19)	− 0.35 (− 1.43, 0.93)

Statistically significant (*p* < 0.05) estimates are marked in bold. Multiple linear and logistic regression models were fitted to assess β-coefficients and OR and their 95%CI for semen quality parameters across blood log-transformed Pb concentrations. Models were adjusted by age (years), body mass index (kg/m^2^), physical activity (MET min/week), and abstinence period (days)

*OR* odds ratio

## Discussion

This study provides valuable insights into the association of metal(loid) exposure and semen quality, comparing petrochemical workers and non-petrochemical controls. The slightly higher concentrations of Cr and Sn in controls than in PWs do not agree with previous studies showing that workers in industrial settings are often exposed to elevated levels of metals, which can accumulate in blood (Li et al. 2023). Additionally, the lower Pb levels in PWs compared to controls were also somewhat unexpected, as Pb is a known environmental contaminant also associated with industrial activities (Kim et al. 2017). This finding may reflect differences in local environmental exposures and lifestyle factors, such as the location of residence or workplace habits. Although the control group may have other non-industrial sources of metal(loid) exposure, such as diet (Lee et al. 2019; Junqué et al., 2022), these values indicate a scenario of very low exposure regardless of the petrochemical occupation, consistent with previous biomonitoring studies conducted in Spain (Esplugas et al. 2020). Finally, despite the low detection frequencies of As and Tl, monitoring these metals remains essential due to the potential health risks associated with long-term, low-level metal exposure (Osorio-Rico et al. 2017; Jomova et al 2011).

The analysis of semen quality parameters reveals several unexpected findings when comparing PWs with the control group. Contrary to expectations based on literature suggesting that occupational exposures generally impair fertility parameters (Cofone et al. 2023), our results indicate that working in the petrochemical industry does not uniformly reduce semen quality. When assessed against the WHO 2021 reference values, PW appeared to have specific vulnerabilities affecting sperm morphology and semen volume. However, the control group showed a higher overall incidence of impaired motility, total sperm count, and vitality, which could be attributed to lifestyle or environmental factors unrelated to occupational or metal(loid) exposure.

The significant negative correlation between physical activity and BMI aligns with existing literature, highlighting the beneficial impact of physical activity on maintaining a healthy body weight, which is crucial for reproductive health (Service et al. 2023). The strong positive correlations between sperm count, semen volume, and both semen concentration and total motility are consistent with the well-established interdependence of these semen quality parameters, as higher sperm concentration is typically associated with improved motility and overall fertility potential (Cooper et al. 2010). Interestingly, the positive correlation between chromium (Cr) and tin (Sn) suggests shared exposure sources, particularly in industrial or occupational settings like petrochemical complexes, where both metals are present (Gorman Ng et al. 2017).

There is extensive literature investigating the association with metal(loid) exposure and semen quality, as summarized in Table 4. Our correlation analysis and regression models revealed a statistically significant positive correlation of Pb with total motility. Although previous studies have linked blood Pb exposure to reduced sperm vitality (Sukhn et al. 2018; Telisman et al. 2000), impaired sperm morphology (Shi et al. 2021; Robins et al. 1997, Telisman et al. 2000), decreased sperm concentration and total sperm count (Alexander et al. 1996; Telisman et al. 2000; Fatima et al. 2010), reduced semen volume (Fatima et al. 2010), and lower total motility (Fatima et al. 2010; Telisman et al. 2000), as well as associations between seminal Pb and sperm motility and concentration (Pant et al. 2003), our findings align with some, but not all, of this literature. Aribarg and Sukcharoen (1996) reported that higher levels of blood Pb did not have a significant effect on the sperm parameters. Similarly, Bonde et al. (2002) found that there was no indication of a linear trend of lower sperm concentration with increasing blood lead levels. Awadalla et al. (2011) found a non-significant reduction in several sperm quality parameters—including total motility—when comparing low Pb exposure vs. high Pb exposure. Calogero et al. (2021) found that Pb seminal plasma levels had a positive association with progressive sperm motility. In turn, Hernández-Ochoa et al. (2005) indicated that blood Pb was not associated with semen quality, suggesting that Pb in semen compartments assesses better the amount of Pb in the reproductive tract. In a longitudinal study, Williams et al. (2022) did not find associations between peripubertal blood Pb level and sperm parameters. Finally, despite Inhorn et al. (2008) found that men reporting occupational exposures were twice as likely to be infertile as unexposed men, infertile men did not have significantly higher whole blood concentrations of Pb compared to fertile men.

**Table 4:** Summary of studies investigating the association between metal(loid) exposure and semen quality parameters

Study design	Metal(loid)/s	Biofluid	Population group	Finding	Reference
Case–control	Pb, Cd, As, Ba, Hg, U	Blood and seminal fluid	Low semen quality vs. high semen quality	We found that participants with low-quality semen had significantly higher Cd and Ba concentrations in the seminal fluid than participants with normal-quality semen. We also observed significant associations between low sperm viability and higher blood Cd and Ba, as well as higher seminal Pb, Cd, Ba, and U. Furthermore, U concentrations in the seminal fluid were associated with increased odds ratios for below-reference progressive sperm motility and normal morphology	Sukhn et al. 2018
Case–control	Pb	Blood	Low Pb exposure vs. high Pb exposure	The mean BLL in the studied subjects was 20.08 μg/dL. 45% of the studied men had BLL ≥ 20 μg/dL. Non-significant reduction in sperm count, impaired sperm motility, and altered sperm morphology were observed in those with BLL ≥ 20 μg/dL compared to those with BLL < 20 μg/dL. Concerning semen flow cytometry analysis, the percentage of haploid sperms was significantly lower among men with BLL ≥ 20 μg/dL (78%) compared to that among those with BLL < 20 μg/dL (87%). A positive significant correlation was observed between BLL and the percentage of diploid sperms	Awadalla et al. 2011
Case–control	Al, As, Ba, Be Ca, Cd, Co, Cr, Cu, Fe, K, Li, Mg, Mn, Mo, Na, Ni, Pb, Sb, Se, Sr, Zn	Blood	High environmental impact vs. low environmental impact	The semen from the “high environmental impact” group showed higher zinc, copper, chromium, and reduced iron levels, as well as reduced sperm motility	Bergamo et al. 2016
Case–control	Pb, Cd	Seminal plasma	Infertile vs. fertile	An increase in lead and cadmium levels was observed in infertile men, and there was a significant negative correlation between cadmium and lead semen concentration with sperm motility and sperm concentration in oligoasthenospermic men	Pant et al. 2003
Case–control	As, Cd, Cu, Mn, Pb, Se, Zn	Blood	Occupational (exposed vs. non-exposed)	Multiple regression analysis showed that men with reported occupational exposures were twice as likely to be infertile as unexposed men. However, none of the subcategories of infertile men (based on semen analysis results) had significantly higher whole blood concentrations of heavy metals when compared to fertile controls. Blood concentrations were well within the range for referent populations of healthy individuals	Inhorn et al. 2008
Case–control	Zn, Cu, Cd, Pb	Blood, plasma, and seminal plasma	Infertile vs. fertile	There was a significant positive relationship between sperm density and seminal plasma zinc concentration in the fertile, but not in the infertile, men	Stanwell-Smith et al. 1983
Case–control	Pb	Blood	Occupational	The median sperm concentration was reduced by 49% in men with blood lead concentration above 50 µg/dL. There was no indication of a linear trend of lower sperm concentration with increasing blood lead values, but threshold slope least square regression identified a blood lead concentration of 44 µg/dL (beta = − 0.037, *F* = 4.35, *p* = 0.038) as a likely threshold	Bonde et al. 2002
Case–control	Pb	Blood	Occupational	Higher levels of blood lead were found to have no significant effect on the sperm variables	Aribarg and Sukcharoen 1996
Case–control	Ca, Mn, Mg, Zn, Cu	Blood and seminal plasma	Infertile vs. fertile	Weak correlations were demonstrated between blood plasma zinc concentrations and sperm count (rs = 0.18), sperm motility (rs = 0.15), and abnormal sperm morphology (rs = 0.13). Zinc and magnesium concentrations in seminal plasma correlated weakly with sperm count (rs = 0.17 and rs = 0.16, respectively), and copper concentrations in blood plasma with motility (rs = 0.25)	Wong et al. 2001
Case–control	Pb, Cd, Cu, Zn	Blood and urine	Infertile vs. fertile	Heavy metal levels were significantly higher in the infertile group compared to controls. A urinary cadmium level was positively associated with abnormal sperm morphology (*r* = 0.225, *p* < 0.05)	Chabchoub et al. 2021
Case–control	Zn	Blood and seminal plasma	Infertile vs. fertile	The geometric means of the seminal plasma zinc concentration were significantly lower in the infertile group compared with those in the fertile group: 183.6 mg/L (range, 63–499) versus 274.6 mg/L (range, 55–420). There were no significant differences in the geometric means of the blood zinc concentration between the two groups. Seminal plasma zinc concentration was significantly correlated with sperm density (*r* = 0.341, *p* < 0.0001), motility (*r* = 0.253, *p* < 0.0001), and viability (*r* = 0.286, *p* < 0.0001)	Chia et al. 2000
Cross-sectional	Pb, Cd, Hg, As, Ni, V, Se	Seminal plasma	General	We found a positive association of seminal plasma cadmium level with lower total count (OR = 4.48, 95%CI 0.25–80), whereas lead (OR = 4.51, 95%CI 0.86–23) and cadmium (OR = 3.45, 95%CI 0.77–16) seminal plasma levels had a positive association with progressive sperm motility	Calogero et al. 2021
Cross-sectional	Cr	Urine	Men attending an andrology laboratory	Multivariate analysis showed a negative association between the urinary concentrations of chromium and progressive motility (β = − 0.014, *p* = 0.040) and total motility (β = − 1.077, *p* = 0.048), while other semen parameters did not show any statistically significant changes	Pokhrel et al. 2020
Cross-sectional	Cr, Co, Cu, Mo, Se, Zn, Sb, As, Ba, Cd, Pb, Tl, Sn, W, U	Urine	General	A positive association was observed between tin and sperm morphology (β = 4.92, *p* = 0.045). Chromium (β = 1.87, *p* = 0.003) and copper (β = − 1.30, *p* = 0.028) were positively and negatively associated with total sperm count, respectively	Branch et al. 2021
Cross-sectional	Zn, Tl, Sr, Sn, Se, Sb, Pb, Ni, Mo, Mn, Mg, Li, Hg, Ga, Fe, Cu, Cs, Cr, Co, Ca, Bi, Be, As, Al	Seminal plasma	Reproductive centers	We observed positive associations of exposure to lithium (Li), zinc (Zn), and magnesium (Mg) with an increased risk of below reference values for progressive motility and total motility using a logistic regression model (*p* < 0.05). Additionally, our results also revealed a significant inverse relationship between aluminum (Al) and both sperm concentration and count, while cobalt (Co) demonstrated a positive association with sperm concentration (*p* < 0.05). Notably, the WQS model indicated a significant positive association between exposure to metal/metalloid mixtures and the risk of abnormal progressive motility (OR: 1.57; 95%CI: 1.10, 2.24) and abnormal total motility (OR: 1.53; 95%CI: 1.06, 2.19), with this association primarily driven by Li, Mg, and Zn	Wen et al. 2024
Cross-sectional	Pb, Hg, Cd, As, Ni, Mo, Zn, Cu, Se, Fe, Mg, Cr, Ca	Blood	General	The results showed that moderate to high level of blood Pb concentration (> 27.19 μg/L) appeared to be negatively associated with sperm morphology (*p* < 0.05); high level of blood Cd concentration (> 1.44 μg/L) was negatively associated with sperm acrosome reaction (*p* < 0.05); Mo was positively associated with semen volume (*p* < 0.05); however, high level of blood Mo concentration (> 13.52 μg/L) was negatively associated with sperm vitality (*p* < 0.05); high level of blood Zn concentration (> 6.20 mg/L) was positively associated with sperm vitality (*p* < 0.05); moderate level of blood Ca concentration (55.92–66.10 mg/L) was positively associated with semen volume (*p* < 0.05); however, lower level of blood Ca concentration (45.90–55.92 mg/L) was negatively associated with sperm morphology (*p* < 0.05)	Shi et al. 2021
Cross-sectional	Cd	Serum	General	Inverse associations between Cd and semen volume (− 0.03 ± 0.007), progressive motility (− 0.01 ± 0.004), and sperm morphology (− 0.04 ± 0.004) were found across the whole group	Li et al. 2016
Cross-sectional	Zn	Serum and semen	General	The seminal zinc concentration was positively related to the total sperm count, sperm concentration, progressive motility, and normal morphology (Spearman’s test: 0.221, 0.286, 0.269, and 0.183, respectively, *p* < 0.001). It was found that the seminal Zn content in men with normal semen quality was higher compared to men with lowered semen quality (means: 6.37 and 5.03 μmol/ejaculate, respectively, *p* < 0.001). Similarly, the semen volume, total sperm count, sperm concentration, progressive motility, and normal morphology in men with the seminal Zn deficiency were lower than in men with the normal seminal Zn content	Osadchuk et al. 2021
Cross-sectional	Pb	Blood	Occupational	Compared with workers with blood lead concentrations less than 15 µg/dL, workers with current blood lead concentrations of 40 µg/dL or more had an increased risk of below normal sperm concentration (odds ratio (OR) 8.2, 95% confidence interval (95%CI 1.2–57.9)) and total sperm count (OR 2.6, 95%CI 0.4–15.7), based on World Health Organisation standards. Independent of current lead exposure, sperm concentration, total sperm count, and total motile sperm count were inversely related to measures of long-term lead exposure. No association was found between lead exposure and measures of sperm motility, sperm morphology, or serum concentrations of reproductive hormones	Alexander et al. 1996
Cross-sectional	Hg	Blood	General	No significant association (*p* > 0.05) was found between blood concentrations of mercury and any of the other measured semen characteristics (semen volume, total sperm count, sperm concentration, morphology, and motility)	Mocevic et al. 2013
Cross-sectional	Cd, Pb, Se, Zn	Blood and seminal plasma	General	A significant inverse correlation was observed between blood cadmium (CdB) levels and sperm density (*r* = − 0.24, *p* < 0.05) in oligozoospermic men (sperm density below 20 million/mL) but not in normospermic men	Xu et al. 1993
Cross-sectional	Pb	Blood and semen	Occupational	Reasonably consistent and significant associations were found between an increased percentage of sperm with abnormal morphology and higher measures of current blood lead. There were no associations of sperm density or sperm count with any of the lead exposure measures	Robins et al. 1997
Cross-sectional	Pb	Blood	Infertility clinics	With increasing blood lead concentration, there was a corresponding increase in the mean semen lead concentration. Reduction of mean semen volume started at a mean blood lead level of > 40 μg/dL. Mean total count of sperm (× 10^6^/mL) started decreasing at blood lead level of > 30 μg/dL, with a very significant reduction of the count at level > 40 μg/dL. At a mean blood lead level > 35 μg/dL, there was a decrease in mean values for total motility and rapid linear motility of sperm	Fatima et al. 2010
Cross-sectional	Pb	Blood and semen	General	PbB was not associated with semen quality, suggesting that Pb in semen compartments better assesses the amount of Pb in the reproductive tract	Hernández-Ochoa et al. 2005
Cross-sectional	Pb, Cd, Zn, Cu	Blood, serum, and seminal plasma	Occupational	Significant (*p* < 0.05) correlations of BPb with reproductive parameters indicated a Pb-related decrease in sperm density; in counts of total, motile, and viable sperm; and in the percentage and count of progressively motile sperm and an increase in abnormal sperm head morphology. These associations were confirmed by the results of multiple regression, which also showed significant (*p* < 0.05) influence of BCd, SZn, and SCu on certain reproductive parameters. These effects were mainly of lower rank and intensity as compared to Pb-related reproductive effects, whereas BCd contributed to a decrease in sperm motility and an increase in abnormal sperm morphology. The overall study results indicate that even moderate exposures to Pb (BPb < 400 µg/L) and Cd (BCd < 10 µg/L) can significantly reduce human semen quality without conclusive evidence of impairment of male reproductive endocrine function	Telisman et al. 2000
Cross-sectional	Cd	Blood and semen	General	A significant positive correlation was found between cadmium blood levels, number of immotile spermatozoa, and teratozoospermia index (TZI). Significant inverse relationships between cadmium blood concentration and type A and type A + B motility were found	De Franciscis et al. 2015
Cross-sectional	Ca, Mn, Zn, Cu	Blood and semen	General	Seminal Zc and Cu had a relationship with sperm motility	Salsabili et al. 2009
Cross-sectional	Cu, Zn	Serum	General	Progressive motility showed differences among the five copper groups, but multiple logistic analyses did not show that higher or lower serum copper levels had a significant effect on sperm quality. When serum zinc concentration was low, the risk of asthenozoospermia was higher. The ratio of Cu/Zn was higher in the progressive motility abnormal group than in the normal group	Yuyan et al. 2008
Cross-sectional	Cu, Zn	Blood and seminal plasma	Infertile	A significant (*p* < 0.01) inverse correlation was observed between serum Zn and sperm counts	Akinloye et al. 2011
Longitudinal	Pb	Blood	General	Men with peripubertal BLL ≥ 5 µg/dL had significantly lower ejaculated volume than those with BLL < 5 µg/dL (mean = 2.42 vs. 2.89 mL, *p* = 0.02), but this difference was attenuated in adjusted models (mean = 2.60 vs. 2.83 mL, *p* = 0.25). No associations were observed between BLL measured at age 8–9 years and sperm parameters, including sperm concentration, total count, progressive motility, and total progressive motile sperm count, or with the probability of having low semen quality based on sperm count/motility	Williams et al. 2022

Although our study only focuses on the associations between Pb and semen quality parameters, previous research highlights the impact of various metals on male reproductive health. For instance, Pokhrel et al. (2020) found a negative association between urinary chromium concentrations and both progressive and total motility. Bergamo et al. (2016) reported that elevated Zn, Cu, and Cr levels, alongside reduced Fe levels, were linked to decreased sperm motility. De Franciscis et al. (2015) demonstrated a significant positive correlation between blood Cd levels and the number of immotile spermatozoa and a significant inverse relationship between Cd levels and sperm motility, while Chabchoub et al. (2021) found that urinary Cd was positively associated with abnormal sperm morphology. In turn, Li et al. (2016) found inverse associations between Cd and semen volume, progressive motility, and sperm morphology, but Xu et al. (1993) found a significant inverse correlation between blood Cd levels and sperm density in oligozoospermic men, but not in normospermic men (sperm density above 20 million/mL). Branch et al. (2021) demonstrated a positive association between Sn and sperm morphology and a positive and a negative association between Cr and Cu and total sperm count, respectively. Salsabili et al. (2009) observed correlations between seminal Zn and Cu concentrations and sperm motility, while Yuyan et al. (2008) found that varying serum Cu levels did not significantly impact sperm quality. Osadchuk et al. (2021) demonstrated positive associations between seminal Zn and key semen quality parameters, including sperm count, concentration, motility, and morphology, while Stanwell-Smith et al. (1983) reported a significant positive relationship between sperm density and seminal plasma zinc in fertile men, but not in infertile men. Wong et al. (2001) observed weak correlations between blood plasma Zn concentrations and sperm count, motility, and abnormal morphology, as well as between seminal plasma Zn and Mg levels and sperm count, and between blood plasma copper concentrations and motility. Additionally, Chia et al. (2000) showed that seminal plasma zinc concentrations were significantly lower in infertile men compared to fertile men and that seminal plasma Zn was strongly correlated with sperm density, motility, and viability, though no significant differences in blood Zn levels were observed between the two groups. Akinloye et al. (2011) further reported a significant inverse correlation between serum Zn and sperm counts. Wen et al. (2024) observed that Li, Zn, and Mg were linked to an increased risk of impaired motility, while Al was inversely associated with sperm concentration and count. In contrast, Co showed a positive association with sperm concentration. Lastly, Mocevic et al. (2013) did not observe a significant association between Hg blood concentrations and semen volume, total sperm count, sperm concentration, sperm morphology, and sperm motility.

## Strengths and limitations

This study boasts several strengths, such as employing comprehensive semen quality assessments aligned with the WHO 2021 guidelines and conducting a detailed examination of multiple metal(loid) exposures across both occupational and non-occupational settings. The inclusion of a control group enabled meaningful comparisons, and the application of advanced statistical models strengthened the analysis of relationships between metal exposure and semen quality metrics. Moreover, the correlation network map provided valuable insights into the complex interconnections among sociodemographic factors, lifestyle, and metal(loid) exposure. Nevertheless, there are significant limitations to consider. The relatively small sample size—particularly in the petrochemical worker subgroup due to low participation despite extensive recruitment efforts—may reduce the study’s statistical power and limit the generalizability of the findings. The cross-sectional design further hampers the ability to infer causality between metal(loid) exposure and semen quality outcomes. Additionally, semen quality is known to be influenced by multiple physiological and psychosocial factors, such as age, stress, and sexual behavior, which could not be fully captured within this preliminary design. Although we adjusted our models for major confounders (age, BMI, physical activity, and abstinence period), unmeasured variables such as diet or arousal-related factors might have also played a role. For these reasons, future studies should aim to include a larger number of participants and adopt a longitudinal approach over several years to strengthen causal inference and confirm the patterns observed in the present study.

## Conclusions

This study provides important insights into the associations between metal(loid) exposure and semen quality among petrochemical workers compared to a non-petrochemical control group. Contrary to the common expectation that occupational exposure in petrochemical industries universally impairs semen quality, our findings indicate a more complex relationship. Specifically, while PWs exhibited higher sperm concentration and generally better semen parameters, the control group demonstrated a greater prevalence of impaired motility, sperm count, and vitality. These surprising results imply that non-occupational factors, such as lifestyle or other environmental exposures unrelated to metal(loid)s, may play a crucial role in influencing semen quality among the controls. Furthermore, although our analysis found no significant negative associations between Pb exposure and most semen quality parameters, the positive association between blood Pb levels and total motility warrants further investigation. While this is not the first study to report such an opposing association, it challenges some established views on the reproductive toxicity of Pb and emphasizes the need for more detailed studies that account for various sources and forms of metal exposure.

Overall, our study highlights the necessity for continued monitoring of metal(loid) exposure in occupational settings and the importance of considering non-occupational factors in reproductive health research. Future studies should explore the mechanisms underlying the differential impact of metal(loid)s on semen quality and assess other potential environmental and lifestyle contributors to reproductive health in exposed populations.

## Supplementary Information

Below is the link to the electronic supplementary material.

Supplementary file 1 (DOCX 85.5 KB)

## Data Availability

The datasets analyzed in the current study are available in the Supporting Information.
