# Supplementary material for: Exploring the association between metal(loid)s and human semen quality: a preliminary case study in a petrochemical complex
Source: Environ Sci Pollut Res Int. 2025 Nov 14;32(46):26207–29. doi: 10.1007/s11356-025-37173-x (PMC12672805; doi:10.1007/s11356-025-37173-x)
Supplement: Supplementary file 1 — (DOCX 85.5 KB) [file 11356_2025_37173_MOESM1_ESM.docx]

**Exploring the association between metal(loid)s and human semen quality: A preliminary case study in the surroundings of a petrochemical complex**

Elena Sánchez-Resino^1,2,3^, Ana González-Ruiz^1,2,3^, Jordi Sierra^2,4^, Carlos Martínez-Pinto^5^, María Fernández de la Puente ^6,7,8^, Nadine Alkhoury ^6,7,8^, María Ángeles Martínez ^6,7,8,9^, Nancy Babio^6,7,8^, Albert Salas-Huetos^8,10^, Jordi Salas-Salvadó^6,7,8^, Rubén Gil-Solsona^5^, Pablo Gago-Ferrero^5^, José L. Domingo^1,2,3^, Montse Marquès^1,2,3,5^.

^1^ Universitat Rovira i Virgili, Laboratory of Toxicology and Environmental Health, Faculty of Medicine and Health Science, Reus, Catalonia, Spain.

^2^ Center of Environmental, Food and Toxicological Technology - TecnATox, Universitat Rovira i Virgili, Reus, Catalonia, Spain.

^3^ Institut d’Investigació Sanitària Pere Virgili, Reus, Catalonia, Spain.

^4^ Faculty of Pharmacy, Barcelona University, Avda Joan XXIII s/n, 08028 Barcelona, Spain.

^5^ Institute of Environmental Assessment and Water Research (IDAEA-CSIC), Carrer Jordi Girona 18-26, Barcelona, 08034, Spain.

^6^ Universitat Rovira i Virgili, Departament de Bioquímica i Biotecnologia, Alimentació, Nutrició, Desenvolupament i Salut Mental ANUT-DSM, Reus, Spain.

^7^ Institut d'Investigació Sanitària Pere Virgili (IISPV), Reus, Spain.

^8^ CIBER de Fisiopatología de la Obesidad y Nutrición, Instituto de Salud Carlos III.

^9^ Universitat Autònoma de Barcelona, Department of Pharmacology, therapeutics and Toxicology, Faculty of veterinary. Cerdanyola del Vallès, 08193, Catalonia, Spain.

^10^ Universitat Rovira i Virgili, Unitat de Medicina Preventiva i Bioestadística, Faculty of Medicine and Health Science, Reus, Catalonia, Spain.

**Corresponding author:**

Montse Marquès

[montserrat.marques@urv.cat](mailto:montserrat.marques@urv.cat)

**Supporting Information**

**Table S1.** Demographic, anthropometric and lifestyle characteristics, blood metal(loid) levels and semen quality parameters.

| **Occupational group** | **Age** (years) | **BMI**  (Kg/m^2^) | **Physical activity** (METs/week) | **Concentration** (x10^6) | **Normal form** (%) | **Sperm count** (x10^6) | **Total motility** (%) | **Vitality (%)** | **Volume** (mL) | **As** | **Cr** | **Pb** | **Sn** | **Tl** |
| --- | --- | --- | --- | --- | --- | --- | --- | --- | --- | --- | --- | --- | --- | --- |
| PW | 36 | 30.5 | 4521 | 96.8 | 15.5 | 290.4 | 54 | 71 | 3 | <LOD | <LOD | 26.08 | <LOD | <LOD |
| PW | 32 | 26.9 | 3142 | 154.75 | 21 | 619 | 54 | 70 | 4 | <LOD | <LOD | <LOD | <LOD | <LOD |
| PW | 30 | 24.1 | 6732 | 31.8 | 20 | 213.06 | 56.5 | 76 | 6.7 | <LOD | <LOD | <LOD | <LOD | <LOD |
| PW | 32 | 21.8 | 5245 | 79.25 | 25.5 | 126.8 | 77 | 82.5 | 1.6 | <LOD | <LOD | <LOD | <LOD | <LOD |
| PW | 36 | 24.6 | 2130 | 69.5 | 19 | 430.9 | 59.3 | 80 | 6.2 | <LOD | <LOD | <LOD | <LOD | <LOD |
| PW | 32 | 22.4 | 2965 | 206.5 | 30.5 | 826 | 65 | 73 | 4 | <LOD | 30.06 | <LOD | <LOD | <LOD |
| PW | 36 | 23.9 | 1473 | 172 | 2 | 258 | 54 | 80 | 1.5 | <LOD | <LOD | 30.36 | <LOD | <LOD |
| PW | 31 | 23.8 | 1613 | 198.75 | 7.5 | 795 | 71.5 | 83 | 4 | <LOD | <LOD | 24.71 | <LOD | <LOD |
| PW | 26 | 24.9 | 3511 | 127.15 | 4.5 | 139.86 | 45.5 | 75 | 1.1 | <LOD | <LOD | <LOD | <LOD | <LOD |
| PW | 32 | 28.4 | 4625 | 64.25 | 4 | 257 | 61.5 | 81 | 4 | <LOD | <LOD | <LOD | <LOD | <LOD |
| PW | 36 | 23 | 2625 | 219.5 | 8 | 702.4 | 56.5 | 91 | 3.2 | <LOD | <LOD | <LOD | <LOD | <LOD |
| PW | 35 | 22.6 | 3282 | 42 | 22.5 | 84 | 67.5 | 79.5 | 2 | <LOD | <LOD | 12.54 | <LOD | <LOD |
| PW | 37 | 24.4 | 1865 | 35.5 | 12.5 | 124.25 | 70.5 | 84 | 3.5 | <LOD | <LOD | 23.05 | <LOD | <LOD |
| PW | 32 | 30.1 | 2741 | 169.25 | 6 | 558.52 | 75.5 | 82.5 | 3.3 | <LOD | <LOD | <LOD | <LOD | <LOD |
| PW | 30 | 24.6 | 5530 | 51.24 | 2 | 128.1 | 41.53 | 84 | 2.5 | <LOD | <LOD | <LOD | <LOD | <LOD |
| PW | 37 | 25.1 | 420 | 11.69 | 2 | 35.07 | 39 | 43 | 3 | <LOD | <LOD | <LOD | <LOD | <LOD |
| PW | 30 | 24.8 | 1329 | 85.57 | 9.5 | 299.5 | 77.17 | 79.5 | 3.5 | <LOD | <LOD | <LOD | <LOD | <LOD |
| PW | 30 | 25.3 | 3573 | 10.05 | 12 | 55.28 | 48.44 | 82 | 5.5 | <LOD | <LOD | <LOD | <LOD | <LOD |
| PW | 34 | 26.8 | 3778 | 58.47 | 21 | 245.57 | 44.89 | 76 | 4.2 | <LOD | <LOD | <LOD | <LOD | <LOD |
| PW | 24 | 26.4 | 5259 | 31.7 | 4 | 79.25 | 70.65 | 87.5 | 2.5 | <LOD | <LOD | 18.32 | 11.85 | <LOD |
| PW | 26 | 22.6 | 5333 | 76.37 | 18.5 | 267.3 | 60.51 | 89.5 | 3.5 | <LOD | 17.34 | 35.17 | 19.72 | <LOD |
| PW | 33 | 25.6 | 5257 | 30.71 | 1 | 184.26 | 50.24 | 92 | 6 | <LOD | <LOD | <LOD | <LOD | <LOD |
| NPW | 24 | 22.4 | 1156 | 18.18 | 7.5 | 90.9 | 71.5 |  | 5 | <LOD | <LOD | 44.06 | <LOD | <LOD |
| NPW | 27 | 23.8 | 1684 | 33 | 3 | 132 | 62 | 72 | 4 | <LOD | <LOD | 51.04 | <LOD | <LOD |
| NPW | 24 | 28 | 1389 | 55.5 | 5.5 | 260.85 | 40.8 | 59 | 4.7 | <LOD | <LOD | 50.19 | <LOD | <LOD |
| NPW | 39 | 24.1 | 4685 | 26.75 | 1.5 | 88.28 | 47.5 | 71 | 3.3 | <LOD | <LOD | 29.4 | <LOD | <LOD |
| NPW | 25 | 22.7 | 4476 | 16.42 | 15 | 43.51 | 50.6 | 76 | 2.65 | <LOD | <LOD | <LOD | <LOD | <LOD |
| NPW | 30 | 25.9 | 2744 | 32.8 | 13 | 180.4 | 59.85 | 74 | 5.5 | <LOD | <LOD | 46.75 | <LOD | <LOD |
| NPW | 36 | 22.8 | 4268 | 73.75 | 6 | 331.88 | 74.9 | 69 | 4.5 | <LOD | <LOD | 72.3 | <LOD | <LOD |
| NPW | 25 | 24.9 | 1965 | 28.5 | 12.5 | 114 | 79.5 | 80.5 | 4 | <LOD | <LOD | 40.77 | <LOD | <LOD |
| NPW | 24 | 30.5 | 2823 | 58.25 | 25.5 | 203.88 | 74.5 | 83.5 | 3.5 | <LOD | <LOD | 32.83 | <LOD | <LOD |
| NPW | 21 | 21.4 | 1138 | 128.76 | 11.5 | 218.89 | 74.66 | 80 | 1.7 | <LOD | <LOD | <LOD | <LOD | <LOD |
| NPW | 40 | 20.5 | 3639 | 96.24 | 24 | 385 | 59 | 84 | 4 | <LOD | <LOD | 33.9 | <LOD | <LOD |
| NPW | 32 | 25.8 | 6573 | 59 | 14.5 | 206.5 | 70 | 76.5 | 3.5 | <LOD | <LOD | <LOD | 10.7 | <LOD |
| NPW | 33 | 27.9 | 6294 | 160.5 | 18.5 | 642 | 84 | 81 | 4 | <LOD | <LOD | 73.91 | <LOD | <LOD |
| NPW | 35 | 26.3 | 504 | 53.5 | 4.5 | 203.3 | 46 | 79 | 3.8 | 15.42 | <LOD | 25.14 | <LOD | <LOD |
| NPW | 23 | 20.9 | 3776 | 110.25 | 17 | 275.63 | 44.2 | 78.5 | 2.5 | <LOD | <LOD | 23.64 | <LOD | <LOD |
| NPW | 28 | 25.6 | 3855 | 26.77 | 5 | 64.25 | 57.82 | 74 | 2.4 | <LOD | <LOD | <LOD | <LOD | <LOD |
| NPW | 24 | 36.5 | 1119 | 51.08 | 6.5 | 102.16 | 47.44 | 72.5 | 2 | <LOD | <LOD | <LOD | <LOD | <LOD |
| NPW | 28 | 23.6 | 1958 | 141.25 | 4.5 | 339 | 66.5 | 80 | 2.4 | <LOD | <LOD | <LOD | <LOD | <LOD |
| NPW | 29 | 21.1 | 7536 | 41.25 | 24 | 288.75 | 69 | 71.5 | 7 | <LOD | <LOD | <LOD | <LOD | <LOD |
| NPW | 23 | 22.4 | 5049 | 91.5 | 22 | 411.75 | 78.5 | 83.5 | 4.5 | 22.66 | <LOD | 14.1 | <LOD | <LOD |
| NPW | 24 | 22.2 | 280 | 66.5 | 13 | 332.5 | 74.5 | 73 | 5 | <LOD | <LOD | 46.79 | <LOD | <LOD |
| NPW | 26 | 25.7 | 5876 | 86 | 14 | 258 | 75.5 | 72.5 | 3 | <LOD | <LOD | 81.29 | <LOD | <LOD |
| NPW | 29 | 23.4 | 2548 | 54.5 | 18 | 272.5 | 81 | 76 | 5 | <LOD | <LOD | <LOD | <LOD | <LOD |
| NPW | 40 | 24 | 1270 | 227.25 | 14.5 | 1022.62 | 59.6 | 86.5 | 4.5 | <LOD | <LOD | 56.08 | <LOD | <LOD |
| NPW | 22 | 25.2 | 2033 | 92 | 7 | 312.8 | 71.5 | 79 | 3.4 | <LOD | <LOD | 42.81 | 9.42 | <LOD |
| NPW | 33 | 25.9 | 1445 | 63.07 | 1.99 | 283.82 | 53.85 | 63.5 | 4.5 | 19.6 | <LOD | <LOD | <LOD | <LOD |
| NPW | 28 | 19.6 | 4633 | 203.25 | 12.5 | 406.5 | 72.5 | 76 | 2 | <LOD | <LOD | <LOD | <LOD | <LOD |
| NPW | 26 | 30 | 1678 | 16.75 | 11 | 83.75 | 52.5 | 72.5 | 5 | <LOD | <LOD | <LOD | <LOD | <LOD |
| NPW | 26 | 19.5 | 8559 | 58.75 | 14 | 158.63 | 41.5 | 87 | 2.7 | <LOD | <LOD | <LOD | <LOD | <LOD |
| NPW | 36 | 23.8 | 5758 | 96.5 | 19 | 347.4 | 79 | 82.5 | 3.6 | <LOD | <LOD | <LOD | <LOD | <LOD |
| NPW | 28 | 23.1 | 2531 | 47.5 | 18.5 | 337.25 | 66.5 | 47 | 7.1 | <LOD | <LOD | 32.53 | <LOD | <LOD |
| NPW | 29 | 21.9 | 9324 | 125.25 | 8 | 688.88 | 74 | 76.5 | 5.5 | <LOD | <LOD | 13.01 | <LOD | <LOD |
| NPW | 29 | 23.9 | 7650 | 84.58 | 16.5 | 422.9 | 61.54 | 68 | 5 | <LOD | <LOD | <LOD | <LOD | <LOD |
| NPW | 21 | 21.8 | 3963 | 86 | 7 | 249.4 | 68.5 | 74 | 2.9 | <LOD | <LOD | 39.48 | <LOD | <LOD |
| NPW | 29 | 28.4 | 2239 | 79.25 | 3 | 47.55 | 75 | 87.5 | 0.6 | <LOD | <LOD | 69.47 | <LOD | <LOD |
| NPW | 33 | 24.9 | 627 | 95.75 | 8 | 383 | 69 | 81 | 4 | <LOD | <LOD | 50.46 | <LOD | <LOD |
| NPW | 25 | 24.1 | 4140 | 61.75 | 7 | 98.8 | 72.5 | 76.5 | 1.6 | <LOD | <LOD | 47.95 | <LOD | <LOD |
| NPW | 33 | 28.1 | 2601 | 31.25 | 5.5 | 53.13 | 51 | 86 | 1.7 | <LOD | <LOD | <LOD | <LOD | <LOD |
| NPW | 21 | 23.5 | 4065 | 37.5 | 4 | 135 | 77.5 | 80 | 3.6 | <LOD | <LOD | 15 | <LOD | <LOD |
| NPW | 24 | 26.4 | 10065 | 28.74 | 3 | 103.46 | 46.41 | 80.5 | 3.6 | <LOD | <LOD | <LOD | <LOD | <LOD |
| NPW | 35 | 21.6 | 5981 | 223.75 | 21.5 | 671.25 | 74 | 78 | 3 | <LOD | <LOD | <LOD | <LOD | <LOD |
| NPW | 21 | 26.9 | 4219 | 71 | 5 | 198.8 | 80 | 77 | 2.8 | <LOD | <LOD | 43.67 | <LOD | <LOD |
| NPW | 20 | 18.7 | 5308 | 62.5 | 0.5 | 312.5 | 49 | 50.5 | 5 | <LOD | <LOD | 37.53 | <LOD | <LOD |
| NPW | 20 | 22.7 | 3373 | 115.25 | 12.5 | 230.5 | 78.5 | 76 | 2 | <LOD | <LOD | 18.44 | 6.34 | <LOD |
| NPW | 25 | 21 | 434 | 3.5 | 11.11 | 5.95 | 49 | 81.5 | 1.7 | <LOD | <LOD | 27.53 | <LOD | <LOD |
| NPW | 29 | 25 | 7207 | 42.25 | 5 | 152.1 | 83.5 | 76 | 3.6 | <LOD | <LOD | <LOD | <LOD | <LOD |
| NPW | 25 | 20.9 | 3378 | 14.5 | 10 | 79.78 | 80 | 72 | 5.5 | <LOD | <LOD | <LOD | <LOD | <LOD |
| NPW | 37 | 23 | 4578 | 29 | 2.5 | 145 | 67.5 | 62.5 | 5 | <LOD | <LOD | <LOD | <LOD | <LOD |
| NPW | 25 | 22.8 | 3554 | 68.16 | 3 | 245.38 | 47.98 | 80 | 3.6 | <LOD | <LOD | <LOD | <LOD | <LOD |
| NPW | 27 | 24.4 | 1613 | 67.83 | 6.93 | 373.07 | 72.33 | 77.5 | 5.5 | <LOD | <LOD | <LOD | <LOD | <LOD |
| NPW | 34 | 21.2 | 1667 | 137.25 | 5 | 384.3 | 69.5 | 83.5 | 2.8 | <LOD | <LOD | 18.45 | <LOD | <LOD |
| NPW | 24 | 19.3 | 1734 | 30 | 4 | 150 | 72 | 62.5 | 5 | <LOD | <LOD | <LOD | <LOD | <LOD |
| NPW | 32 | 19.9 | 3013 | 69 | 4.5 | 393.3 | 61 | 73.5 | 5.7 | <LOD | <LOD | <LOD | <LOD | <LOD |
| NPW | 40 | 22.8 | 4200 | 47.63 | 4 | 242.91 | 61.66 | 79.5 | 5.1 | <LOD | <LOD | 23.76 | <LOD | <LOD |
| NPW | 23 | 24.8 | 5315 | 68.98 | 11.5 | 110.37 | 55.38 | 84 | 1.6 | <LOD | <LOD | <LOD | <LOD | <LOD |
| NPW | 20 | 22.6 | 4266 | 33.5 | 5 | 147.4 | 77.5 | 75.5 | 4.4 | <LOD | <LOD | <LOD | <LOD | <LOD |
| NPW | 21 | 30.1 | 606 | 46.25 | 10.5 | 231.25 | 77.5 | 65.5 | 5 | <LOD | <LOD | <LOD | <LOD | <LOD |
| NPW | 28 | 19.9 | 3245 | 41.75 | 5.5 | 187.88 | 62 | 85 | 4.5 | <LOD | <LOD | <LOD | <LOD | <LOD |
| NPW | 31 | 19.9 | 3865 | 43.5 | 5 | 213.15 | 78.5 | 85 | 4.9 | <LOD | <LOD | 23.39 | <LOD | <LOD |
| NPW | 26 | 24.7 | 1191 | 103.29 | 6.5 | 413 | 74 | 85.5 | 4 | <LOD | <LOD | <LOD | <LOD | <LOD |
| NPW | 26 | 29.2 | 1160 | 56.25 | 1.5 | 348.75 | 61.5 | 80.5 | 6.2 | <LOD | <LOD | <LOD | <LOD | <LOD |
| NPW | 35 | 24.3 | 2816 | 68.65 | 10 | 144.17 | 63.19 | 75.5 | 2.1 | <LOD | <LOD | 15.02 | <LOD | <LOD |
| NPW | 37 | 31.2 | 578 | 52.06 | 21.39 | 119.74 | 73.7 | 80 | 2.3 | <LOD | <LOD | 13.04 | <LOD | <LOD |
| NPW | 31 | 26.2 | 1678 | 21.68 | 2.5 | 108.4 | 72.53 | 63.5 | 5 | <LOD | <LOD | <LOD | <LOD | <LOD |
| NPW | 26 | 24.4 | 7012 | 35.48 | 3.4 | 127.73 | 64.31 | 85 | 3.6 | <LOD | <LOD | <LOD | <LOD | <LOD |
| NPW | 26 | 28.5 | 671 | 25.07 | 15.5 | 102.79 | 28.8 | 77 | 4.1 | <LOD | <LOD | <LOD | <LOD | <LOD |
| NPW | 26 | 20.2 | 3040 | 60.28 | 3 | 72.34 | 46.52 | 77 | 1.2 | <LOD | <LOD | <LOD | <LOD | <LOD |
| NPW | 29 | 22.8 | 7538 | 13.47 | 22 | 43.1 | 64.86 | 87 | 3.2 | 20.14 | <LOD | 15.81 | <LOD | <LOD |
| NPW | 22 | 19.7 | 6900 | 37.77 | 16.5 | 135.97 | 60.47 | 80.5 | 3.6 | <LOD | <LOD | <LOD | <LOD | <LOD |
| NPW | 33 | 26.9 | 965 | 199.06 | 17 | 796.24 | 70.42 | 70 | 4 | <LOD | <LOD | 15.43 | <LOD | <LOD |
| NPW | 40 | 25.7 | 3350 | 52.56 | 4 | 231.26 | 41.28 | 78 | 4.4 | <LOD | <LOD | <LOD | <LOD | <LOD |
| NPW | 22 | 23.4 | 9021 | 39.58 | 29 | 75.2 | 66.14 | 81.5 | 1.9 | <LOD | <LOD | <LOD | <LOD | <LOD |
| NPW | 33 | 25.6 | 7506 | 85.24 | 4.5 | 281.29 | 65.06 | 90.5 | 3.3 | <LOD | <LOD | <LOD | <LOD | <LOD |
| NPW | 29 | 22.7 | 7161 | 0.99 | 0 | 2.18 | 18.52 | 0 | 2.2 | <LOD | <LOD | <LOD | <LOD | <LOD |
| NPW | 29 | 25.3 | 1795 | 88.69 | 26.5 | 310.42 | 75.6 | 90 | 3.5 | <LOD | <LOD | <LOD | <LOD | <LOD |
| NPW | 26 | 29.1 | 2294 | 88.85 | 6.5 | 257.67 | 70.1 | 91 | 2.9 | <LOD | <LOD | <LOD | <LOD | <LOD |
| NPW | 33 | 33.3 | 3730 | 13.47 | 11.5 | 35.02 | 50 | 82.5 | 2.6 | <LOD | <LOD | 12.7 | <LOD | <LOD |
| NPW | 26 | 25.9 | 1716 | 91.64 |  | 348.23 | 77.64 | 88.5 | 3.8 | <LOD | <LOD | 15.66 | <LOD | <LOD |
| NPW | 25 | 27.1 | 6168 | 20.91 | 6.5 | 62.73 | 63.98 | 80 | 3 | <LOD | <LOD | <LOD | <LOD | <LOD |
| NPW | 39 | 27.4 | 442 | 30.55 | 21 | 45.83 | 38.12 | 76 | 1.5 | <LOD | 19.95 | <LOD | <LOD | <LOD |
| NPW | 30 | 27.7 | 4476 | 50.26 | 13.5 | 221.14 | 69.44 | 81 | 4.4 | <LOD | <LOD | <LOD | <LOD | <LOD |
| NPW | 22 | 19.4 | 960 | 17.66 | 9.5 | 28.26 | 37.96 | 87.5 | 1.6 | <LOD | <LOD | <LOD | <LOD | <LOD |
| NPW | 20 | 21.5 | 3937 | 240.11 | 6 | 720.33 | 90.41 | 85.5 | 3 | <LOD | <LOD | <LOD | <LOD | <LOD |
| NPW | 37 | 21.7 | 1077 | 32.52 | 5.5 | 130.08 | 54.72 | 64 | 4 | <LOD | <LOD | <LOD | <LOD | <LOD |
| NPW | 33 | 24.6 | 699 | 0 | 0 | 0 | 0 | 50 | 2 | <LOD | <LOD | <LOD | <LOD | <LOD |
| NPW | 31 | 25.8 | 3436 | 101.33 | 8.5 | 202.66 | 72.87 | 72 | 2 | <LOD | <LOD | <LOD | <LOD | <LOD |
| NPW | 36 | 24.7 | 1963 | 27.92 | 1 | 61.42 | 51.67 | 90 | 2.2 | <LOD | <LOD | <LOD | 21.97 | <LOD |
| NPW | 38 | 23.6 | 3595 | 48.12 | 0 | 168.42 | 47.49 | 61 | 3.5 | <LOD | <LOD | 13.13 | <LOD | <LOD |
| NPW | 20 | 24.5 | 5604 | 81.79 | 16.5 | 286.27 | 91.88 | 73.5 | 3.5 | <LOD | <LOD | <LOD | <LOD | <LOD |
| NPW | 23 | 22.7 | 7832 | 18.32 | 7 | 453.3 | 85.61 | 77.5 | 2.5 | <LOD | 41.71 | 46.45 | 27.77 | <LOD |
| NPW | 28 | 25.5 | 2769 | 13.55 | 9.52 | 50.14 | 60.8 | 74.5 | 3.7 | <LOD | <LOD | <LOD | 11.02 | <LOD |
| NPW | 30 | 22.9 | 2876 | 48.29 | 6.5 | 202.82 | 82.82 | 91.5 | 4.2 | <LOD | <LOD | <LOD | <LOD | <LOD |
| NPW | 30 | 20.7 | 4690 | 287.74 | 39.5 | 1007.09 | 71.52 | 91.5 | 3.5 | <LOD | <LOD | 17.73 | 32.79 | <LOD |
| NPW | 25 | 26.7 | 2958 | 33.18 | 18 | 311.89 | 52.75 | 81 | 9.4 | <LOD | <LOD | <LOD | <LOD | <LOD |
| NPW | 29 | 24 | 2071 | 32.85 | 14 | 131.4 | 20.48 | 38 | 4 | <LOD | <LOD | <LOD | <LOD | <LOD |
| NPW | 36 | 28.4 | 1818 | 95.59 | 2 | 219.86 | 85.16 | 85.5 | 2.3 | <LOD | <LOD | <LOD | <LOD | <LOD |
| NPW | 32 | 21.1 | 6480 | 13.27 | 22 | 79.62 | 63.68 | 81 | 6 | <LOD | <LOD | <LOD | <LOD | <LOD |
| NPW | 34 | 25.1 | 1987 | 22.88 | 29.5 | 125.84 | 50 | 85 | 5.5 | <LOD | <LOD | 20.61 | <LOD | <LOD |
| NPW | 30 | 25.1 | 2671 | 28.69 | 4.5 | 114.76 | 56.52 | 61.5 | 4 | <LOD | <LOD | <LOD | <LOD | <LOD |
| NPW | 20 | 26.3 | 701 | 43.52 | 19.5 | 104.45 | 41.94 | 92.5 | 2.4 | <LOD | <LOD | <LOD | <LOD | <LOD |
| NPW | 19 | 22.1 | 4797 | 92.79 | 10.5 | 324.77 | 83.38 | 87 | 3.5 | <LOD | <LOD | <LOD | <LOD | <LOD |
| NPW | 28 | 25.7 | 2626 | 48.29 | 6 | 270.42 | 73.44 | 82.5 | 5.6 | <LOD | <LOD | <LOD | <LOD | <LOD |
| NPW | 38 | 21.7 | 1570 | 161.61 | 15 | 484.83 | 56.2 | 73.5 | 3 | <LOD | <LOD | <LOD | <LOD | <LOD |
| NPW | 23 | 24.8 | 1524 | 8.54 | 12 | 29.89 | 34.8 | 47 | 3.5 | <LOD | <LOD | <LOD | <LOD | <LOD |
| NPW | 23 | 20.3 | 5678 | 47.3 | 11.5 | 118.25 | 44.14 | 82.5 | 2.5 | <LOD | <LOD | <LOD | <LOD | <LOD |
| NPW | 40 | 26.4 | 4233 | 84.91 | 5 | 352.58 | 61.81 | 89 | 4.15 | <LOD | <LOD | <LOD | <LOD | <LOD |
| NPW | 37 | 24.3 | 2619 | 36.3 | 7.5 | 61.71 | 32.47 | 68 | 1.7 | <LOD | <LOD | <LOD | <LOD | <LOD |
| NPW | 31 | 26.8 | 3655 | 36.3 | 13 | 98.01 | 85.17 | 96 | 2.7 | <LOD | <LOD | <LOD | <LOD | <LOD |
| NPW | 22 | 24.7 | 2385 | 97.06 | 15.5 | 145.59 | 78.92 | 92.5 | 1.5 | <LOD | <LOD | <LOD | <LOD | <LOD |
| NPW | 29 | 20.5 | 6573 | 17.52 | 15 | 96.36 | 71.13 | 89 | 5.5 | <LOD | <LOD | <LOD | <LOD | <LOD |
| NPW | 20 | 25.2 | 4224 | 45.99 | 2.5 | 183.96 | 66.37 | 78.5 | 4 | <LOD | <LOD | <LOD | <LOD | <LOD |
| NPW | 23 | 24.8 | 2424 | 34.6 | 4 | 170.8 | 41.31 | 65.5 | 5 | <LOD | <LOD | <LOD | <LOD | <LOD |
| NPW | 31 | 17.4 | 10770 | 31.7 | 19.5 | 158.5 | 62.1 | 85.5 | 5 | <LOD | <LOD | <LOD | <LOD | <LOD |
| NPW | 26 | 26.3 | 6476 | 49.76 | 9.5 | 174.16 | 39.41 | 52.5 | 3.5 | <LOD | <LOD | <LOD | <LOD | <LOD |
| NPW | 27 | 20.7 | 10421 | 8.09 | 6 | 20.23 | 49.76 | 56 | 2.5 | <LOD | <LOD | <LOD | 7.52 | <LOD |
| NPW | 34 | 25.2 | 3851 | 33.34 | 14 | 140.03 | 45.21 | 87 | 4.2 | <LOD | <LOD | <LOD | <LOD | <LOD |
| NPW | 26 | 33.5 | 5138 | 21.46 | 9 | 42.92 | 37.21 | 62.5 | 2 | <LOD | <LOD | 19.88 | <LOD | <LOD |
| NPW | 23 | 28.9 | 2051 | 13.6 | 9.5 | 82.96 | 36.49 | 85.5 | 6.1 | <LOD | <LOD | <LOD | <LOD | <LOD |
| NPW | 22 | 21.9 | 6182 | 48.61 | 4.5 | 106.94 | 84.26 | 94.5 | 2.2 | <LOD | <LOD | 22.27 | <LOD | <LOD |
| NPW | 20 | 24.5 | 1126 | 17.49 | 28 | 55.97 | 49.79 | 79.5 | 3.2 | <LOD | <LOD | <LOD | <LOD | <LOD |
| NPW | 28 | 31.2 | 699 | 91.32 | 2 | 319.62 | 66.43 | 78.5 | 3.5 | <LOD | <LOD | <LOD | <LOD | <LOD |
| NPW | 24 | 24.9 | 3729 | 40.73 | 18.5 | 130.34 | 68.32 | 85.5 | 3.2 | <LOD | <LOD | <LOD | <LOD | <LOD |
| NPW | 27 | 21.1 | 3781 | 65.2 | 5 | 189.08 | 77.08 | 85.5 | 2.9 | <LOD | <LOD | 65.88 | <LOD | <LOD |
| NPW | 30 | 26.9 | 5249 | 63.89 | 5 | 223.62 | 60.87 | 83 | 3.5 | <LOD | <LOD | <LOD | <LOD | <LOD |
| NPW | 29 | 21.3 | 895 | 20.91 | 8 | 127.55 | 52.76 | 85 | 6.1 | 13.07 | <LOD | <LOD | <LOD | <LOD |
| NPW | 31 | 25.1 | 1622 | 61.92 | 18 | 216.72 | 46.65 | 78 | 3.5 | <LOD | <LOD | <LOD | <LOD | <LOD |
| NPW | 32 | 34.4 | 10886 | 39.58 | 8.5 | 55.41 | 41.49 | 88.5 | 1.4 | <LOD | <LOD | <LOD | <LOD | <LOD |
| NPW | 26 | 21.1 | 3049 | 193 | 10 | 38.6 | 60.87 | 95 | 5 | <LOD | <LOD | <LOD | <LOD | <LOD |
| NPW | 29 | 29.2 | 1768 | 31.04 | 2.5 | 142.78 | 51.61 | 91 | 4.6 | <LOD | <LOD | <LOD | <LOD | <LOD |
| NPW | 31 | 22.7 | 1697 | 56.83 | 9.5 | 170.49 | 70.27 | 79.5 | 3 | <LOD | <LOD | <LOD | <LOD | <LOD |
| NPW | 36 | 21.4 | 2000 | 2.91 | 7 | 7.28 | 17.09 | 86 | 2.5 | <LOD | <LOD | <LOD | <LOD | <LOD |
| NPW | 30 | 23.1 | 3656 | 28.25 | 15 | 79.1 | 69.04 | 88.5 | 2.8 | 25.41 | 107.9 | 247.74 | 20.65 | <LOD |
| NPW | 22 | 21.1 | 4340 | 25.4 | 24 | 101.6 | 64.8 | 90 | 4 | <LOD | <LOD | <LOD | <LOD | <LOD |
| NPW | 27 | 25 | 4918 | 18.94 | 13 | 66.29 | 57.81 | 92 | 3.5 | <LOD | <LOD | <LOD | <LOD | <LOD |
| NPW | 32 | 23.1 | 4103 | 217.78 | 1.5 | 479.12 | 46.93 | 95.5 | 2.2 | <LOD | <LOD | <LOD | <LOD | <LOD |
| NPW | 36 | 22.7 | 1030 | 124.66 | 13.5 | 75.55 | 56.87 | 86 | 1.65 | <LOD | <LOD | <LOD | <LOD | <LOD |
| NPW | 27 | 26 | 2551 | 39.42 | 20.5 | 130.09 | 79.55 | 87.5 | 3.3 | <LOD | <LOD | <LOD | <LOD | <LOD |
| NPW | 29 | 21.6 | 5458 | 65.2 | 20.5 | 339.04 | 84.2 | 95 | 5.2 | <LOD | <LOD | <LOD | <LOD | <LOD |
| NPW | 28 | 24.1 | 5883 | 58.47 | 12.5 | 292.35 | 57.33 | 92 | 5 | <LOD | <LOD | <LOD | <LOD | <LOD |
| NPW | 21 | 21.2 | 5521 | 80.8 | 14 | 169.68 | 73.92 | 90 | 2.1 | <LOD | <LOD | <LOD | <LOD | <LOD |
| NPW | 20 | 23.8 | 11012 | 62.25 | 7.5 | 180.53 | 35.31 | 86 | 2.9 | <LOD | <LOD | <LOD | <LOD | <LOD |
| NPW | 25 | 25.4 | 13911 | 21.46 | 7 | 139.49 | 43.81 | 95 | 6.5 | <LOD | <LOD | <LOD | <LOD | <LOD |
| NPW | 34 | 25.1 | 4896 | 70.79 | 24 | 332.71 | 84.36 | 72 | 4.7 | <LOD | <LOD | <LOD | <LOD | <LOD |
| NPW | 18 | 27.8 | 9906 | 46.15 | 12 | 101.53 | 59.35 | 94.5 | 2.2 | <LOD | <LOD | <LOD | <LOD | <LOD |
| NPW | 21 | 25 | 5950 | 11.37 | 8 | 32.97 | 24.77 | 84.5 | 2.9 | <LOD | <LOD | <LOD | <LOD | <LOD |
| NPW | 34 | 24.6 | 5072 | 48.78 | 18.5 | 151.22 | 39.95 | 84.5 | 3.1 | 17.3 | <LOD | <LOD | <LOD | <LOD |
| NPW | 21 | 21.6 | 3615 | 20.8 | 11.5 | 107.12 | 36.7 | 86 | 5.15 | <LOD | <LOD | <LOD | <LOD | <LOD |
| NPW | 34 | 27.5 | 9343 | 3.3 | 0 | 8.75 | 3.92 | 33.5 | 2.65 | <LOD | <LOD | <LOD | <LOD | <LOD |
| NPW | 31 | 20.9 | 10508 | 27.37 | 5 | 117.69 | 49.05 | 91.5 | 4.3 | <LOD | <LOD | <LOD | <LOD | <LOD |
| NPW | 39 | 29.2 | 308 | 15.68 | 4 | 34.5 | 46.98 | 88 | 2.2 | <LOD | <LOD | 18.77 | <LOD | <LOD |
| NPW | 34 | 21.2 | 23357 | 27.92 | 15.42 | 156.35 | 24.71 | 83.5 | 5.6 | <LOD | <LOD | <LOD | <LOD | <LOD |
| NPW | 35 | 21.7 | 4481 | 70.79 | 7.5 | 332.71 | 84.36 | 72 | 4.7 | <LOD | <LOD | 38.03 | <LOD | <LOD |
| NPW | 29 | 27.5 | 10517 | 51.57 | 10 | 60.85 | 71.82 | 93 | 1.18 | <LOD | <LOD | <LOD | <LOD | <LOD |
| NPW | 19 | 26.2 | 1026 | 43.85 | 2.5 | 135.94 | 47.2 | 83 | 3.1 | <LOD | <LOD | <LOD | 8.64 | <LOD |

**Table S2.** Spearman correlation coefficients (ρ) and statistical significance (*p*-value) between sociodemographic, anthropometric and lifestyle factors, metal(oid) concentrations and semen quality parameters.

|  | **Abstinence** | **age** | **BMI** | **Physical activity** | **Concentration** | **Normal form** | **Sperm count** | **Total motility** | **Vitality** | **Volume** | **As** | **Cr** | **Pb** | **Sn** |
| --- | --- | --- | --- | --- | --- | --- | --- | --- | --- | --- | --- | --- | --- | --- |
| **As** | -0.07 | 0.07 | -0.04 | -0.03 | -0.06 | 0.07 | -0.02 | -0.04 | 0.06 | 0.06 | 1.00 | 0.15* | 0.11 | 0.07 |
| **Cr** | -0.09 | 0.03 | -0.05 | 0.02 | -0.03 | 0.16* | 0.05 | 0.03 | 0.01 | -0.10 | 0.15* | 1.00 | 0.15 | 0.38*** |
| **Pb** | 0.17* | 0.05 | -0.05 | -0.11 | 0.16* | 0.01 | 0.17* | 0.24** | -0.09 | -0.04 | 0.11 | 0.15 | 1.00 | 0.17* |
| **Sn** | -0.09 | -0.11 | -0.04 | 0.08 | -0.05 | -0.01 | -0.02 | 0.08 | 0.06 | -0.15 | 0.07 | 0.38*** | 0.17* | 1.00 |

*p<0.05; **p<0.01; ***p<0.001
